# Spiritual health in the reform of spanish renaissance nursing

**DOI:** 10.15649/cuidarte.3893

**Published:** 2024-11-13

**Authors:** Aarón Muñoz-Devesa, José Carlos Bermejo Higuera, José María Galán-González-Serna, Isabel Morales-Moreno

**Affiliations:** 1 Servicio Murciano de Salud, Murcia, España. E-mail: aaronmunozdevesa@gmail.com Servicio Murciano de Salud Murcia España aaronmunozdevesa@gmail.com; 2 Centro Humanización de la Salud, Madrid, España. E-mail: jcbermejo@humanizar.es Centro Humanización de la Salud Madrid España jcbermejo@humanizar.es; 3 Centro Universitario San Juan de Dios, Sevilla, España. E-mail: josemaria.galan@sjd.es Centro Universitario San Juan de Dios Sevilla España josemaria.galan@sjd.es; 4 Universidad Católica de Murcia, Murcia, España. E-mail: imorales@ucam.edu Universidad Católica de Murcia Murcia España imorales@ucam.edu

**Keywords:** Spirituality, Health, Nursing, Nursing History, Validation Study., Espiritualidad, Salud, Enfermería, Historia de la Enfermería, Estudio de Validación, Espiritualidade, Saúde, Enfermagem, História da Enfermagem, Estudo de Validação

## Abstract

**Introduction:**

For decades, spirituality has been rediscovered by health sciences, offering a foundation for how individuals relate to themselves, others, nature, or the transcendent. It is shaped by values and beliefs that guide salutogenic behaviors. However, theoretical development is still necessary, and the history of nursing is a place to find referents.

**Objective:**

To historically validate the concept of spiritual health through the nursing care of St. John of God, the father reformer of nursing within the Hispanic Catholic ambit.

**Materials and Methods:**

Historiographic study based on the works of St. John of God and his mentor, spiritual master, and promoter, St. John of Avila. The categories of analysis were the definition and the four domains of spiritual health following Fisher's model.

**Results-Discussion:**

Comprehensive care of the human being encompassing the four domains was observed. Self: Self-care, introspection, and balance; Others: Compassion, empathy, and humane treatment; Nature: Contact with nature for healing; Transcendence: Religious faith as a source of meaning and peace. For John of God, spiritual health is the complete fulfillment of the individual.

**Conclusions:**

Nursing practices of St. John of God validate the concept of spiritual health within Hispanic nursing. Therefore, we can affirm that his ideas may be relevant to nursing today.

## Resumen.

**Introducción:**

Desde hace décadas, la espiritualidad está siendo redescubierta por las ciencias de la salud, siendo el fundamento del cómo la persona se relaciona consigo misma, con los otros, con la naturaleza o lo transcendente, al estar formada por los valores y creencias que orientan las conductas salutogénicas. No obstante, aún es necesario el desarrollo teórico, siendo la Historia de la Enfermería un lugar donde encontrar referentes.

**Objetivo:**

Realizar una validación histórica del concepto salud espiritual a través de la Enfermería de San Juan de Dios, padre reformador de la Enfermería en el ámbito católico hispánico.

**Materiales y Métodos:**

Se realizó un estudio historiográfico basado en las obras de San Juan de Dios y de su guía, maestro espiritual, e impulsor, San Juan de Ávila, siendo las categorías de análisis la definición y los cuatro dominios de la salud espiritual siguiendo el modelo de Fisher.

**Resultados-Discusión:**

Se observa un cuidado integral del ser humano que abarca los cuatro dominios; Yo: Autocuidado, introspección y equilibrio; Otros: Compasión, empatía y trato humano; Naturaleza: Contacto con la naturaleza para la sanación; Trascendencia: Fe religiosa como fuente de significado y paz. Para Juan de Dios la salud espiritual es la realización plena de la persona.

**Conclusiones:**

La Enfermería de San Juan de Dios valida el concepto de salud espiritual en la enfermería hispánica. Por ello, podemos afirmar que sus ideas pueden ser relevantes para la Enfermería actual.

## Resumo.

**Introdução:**

Há décadas, a espiritualidade vem sendo redescoberta pelas ciências da saúde, sendo a base de como a pessoa se relaciona consigo mesma, com os outros, com a natureza ou com o transcendente, sendo forma da pelos valores e crenças que orientamos comportamentos saluto gênicos. Entretanto, ainda há necessidade de desenvolvimento teórico, e a história da enfermagem é um lugar onde se pode encontrar referências.

**Objetivo:**

Realizar uma validação histórica do conceito de saúde espiritual por meio da Enfermagem de São João de Deus, o pai reformador da Enfermagem no âmbito católico hispânico.

**Materiais e Métodos:**

Foi realizado um estudo historiográfico com base nas obras de São João de Deus e de seu guia, mestre espiritual e promotor, São João de Ávila, tendo como categorias de análise a definição e os quatro domínios da saúde espiritual segundo o modelo de Fisher.

**Resultados-Discussão:**

Observamos um cuidado integral do ser humano que engloba os quatro domínios; Eu: autocuidado, introspecção e equilíbrio; Outros: compaixão, empatia e tratamento humano; Natureza: contato com a natureza para a cura; Transcendência: fé religiosa como fonte de significado e paz. Para João de Deus, a saúde espiritual é a plena realização da pessoa.

**Conclusões:**

A enfermagem de São João de Deus valida o conceito de saúde espiritual na enfermagem hispânica. Portanto, podemos afirmar que suas ideias podem ser relevantes para a enfermagem atual.

## Introduction

According to the Nursing Outcomes Classification (NOC), spiritual health consists of

*"connectedness with self, others, higher power, all life, nature, and the universe that transcends and encompasses self*,"

^1^^)^ alluding to the four domains of spiritual well-being described by Fisher^2^, that is, the relationship the person has with self, others, nature, or the transcendent world. However, although spirituality has been revalued as a salutogenic factor in recent decades, there is still a lack of consensus and a search for references that facilitate the integration of spirituality in the different social and health professions since the biological paradigm is still maintained, as opposed to a more integrative one, which is why a search for references in the past could be useful to validate these concepts, as well as to reinforce attention to the suffering that can be caused by neglecting this dimension of the person^3^^, ^^7^.

Today, nursing theoretical development expresses the relevance of spirituality, both in its taxonomies and theories and models. However, this is not limited to the 21st century. It has been considered for centuries, especially at the dawn of the profession, back in the 16th century, when the foundations of the profession were developed by whom we could consider the father of Hispanic nursing, St. John of God, and his disciples. Their influence gave rise to other nursing care orders that expanded throughout the world of Catholic scope, such as the Obregonians, Bethlemites, Hippolytes, or the Daughters of Charity, all of which integrated spiritual health care into their constitutions and rules, as well as in their writings. Despite being distinct schools, these groups shared a common model -what we could call the model of hospitality- based on the 14 works of mercy: 7 corporal and 7 spiritual. Certainly, Florence Nightingale established the professional-scientific bases of contemporary nursing; however, her work should be considered a reform and development of an earlier model, which she herself acknowledged in her writings. Therefore, indebted to this Hispanic nursing, this article focuses on such nursing, specifically on the contributions of St. John of God, who still keeps alive the spirit of professional conscience today^8^.

At the beginning of the 16th century in Spain, after experiencing different trades and personal crises, St. John of God discovered his calling to care for the most vulnerable of that time when he came into contact with the great spiritual leader of the time, St. John of Avila, who would guide St. John of God’s spiritual growth and support his nursing work to reform social and healthcare in Granada and across Spain. Thus, in 1537, he began his wanderings, culminating his work in the creation of a hospital that would serve as a model for future institutions that would be created or managed by the new nursing structured by him and his disciples. St. John of God died in 1550, the year that began what can be called the golden age of Spanish nursing, compared to the dark period of nursing in the Protestant world^9^. Illustrious figures such as Antón Martín, Pedro Pecador, Bernardino de Obregón, Andrés Fernández, and Simón López took the reins of the Spanish nursing reform of St. John of God, even advancing it further by the establishment of the first nursing schools. They authored books by nurses for nurses, such as *Instrucción de Enfermeros* (Instruction for nurses), considered possibly the first nursing book; they also edited the *Tratado de lo que se ha de hacer con aquellos que están en el artículo de la muerte* (Treatise on what to do with those who are at the point of death), a manual for providing care to the dying, aimed at nurses specializing in end-of-life care^8^.

Therefore, as a first approach to a possible historical validation of the NOC spiritual health and the domains of spiritual well-being developed by Fisher in the aforementioned hospitality model, this study aims to describe spiritual health in St. John of God’s nursing, influenced by his mentor, spiritual master, and great promoter of his work, St. John of Avila, through the current definition and Fisher's four domains. This allows us to validate these elements of current nursing and find references from the past that strengthen spiritual care and its related concepts.

## Materials and Methods

To this end, a historiographical study was conducted between 2020 and 2022, following the history of mentalities approach with a particular focus on certain aspects of the modern biographical method to understand the influence of the context on a specific person and vice versa, in a cyclical and circular manner^10^^, ^^11^.

The primary and secondary sources used were the following:

- The first biography of St. John of God written by Castro^12^
- The process of the beatification of St. John of God^13^
- Letters of St. John of God^14^
- The judicial process of the lawsuit for the ownership of St. John of God's Hospital^15^
- St. John of Avila's epistolary, which includes three letters sent to St. John of God^16^
- Audi, Filia (Listen, O Daughter) by St. John of Avila^17^

After gathering the sources, we conducted the content analysis, using Fisher's model^2^ to extract a priori categories of analysis:

- Spiritual health: Self-awareness, beliefs, values, meaning of life, objectives, goals etc.
- Self: Self-awareness, beliefs, values, objectives, goals, etc.
- Others: Interpersonal relationships, capacity to love, empathy, compassion, etc.
- Nature: Sense of connection with nature, respect for the environment, etc.
- Transcendence: Religious beliefs, spirituality, sense of sacredness, etc.

Once the information was categorized, the fragments were critically compared by topic, triangulating the information obtained between texts, previous studies, and the researcher's assumptions to ensure the validity and reliability of the findings, which are freely available and searchable in Mendeley Data^18^.

## Results - Discussion

### 1. Spiritual health

Regarding the definition of the concept, St. John of God’s master, St. John of Avila, argues that spiritual health is the state of innocence in which a person experiences a sense ofwholeness within themselves and with life as a whole:

*"... perfect concord with one and other, within themselves, and with God, living in the quiet state of innocence, with their sensibility obeying reason, and reason obeying God; and so they were at peace with God, and understood themselves very well, and had peace with each other"* ^17^

In the case of John of God, we do not observe a theoretical definition, but his actions demonstrate his approach: he went through the bodies to the souls to obtain this health outcome. This can be seen in the clinical cases that Castro presents in his biography, where no one ever dies without finding peace or being guided towards it^12^. Nevertheless, in his various crises, he expressed his desire to find "peace and quiet for this soul"^12^ and this could be his ultimate definition of spiritual health.

However, both the master and his disciple encounter three stressors that hinder this subtype of health: the world, the devil, and the flesh. In one of his letters, John of God writes:

*"We are, if we see it rightly, in a constant war with the world, the devil and the flesh, and it is always necessary for us to take care of ourselves"* ^14^

Today, we might refer to them as hedonism, individualism, materialism, and over-identification with mental judgments since these elements interfere with the areas of spiritual health and separate individuals from their authentic selves, others, the environment, and the transcendent. Through spiritual development, spiritual health can be achieved by following the path outlined by Avila, which moves from "*non-being*" to *"being,*" then from "*being*" to "*good being*," ultimately leading to the Beatitude -the supreme expression of happiness in Renaissance Spain, as an expression of the fullness of the person.

### 1.1 Relationship with the inner self

Before becoming a nurse, John of God was on a journey of self-discovery through various crises or kenosis, as Sánchez^19^ refers. He was searching for meaning, for answers to fundamental questions about himself through his values and beliefs, as this domain addresses the question "Who I am?" shaping one's self-awareness, self-esteem, and self-fulfillment as an individual^20^. Therefore, after meeting and beginning the spiritual guidance of St. John of Avila, St. John of God came to understand who he was, his vocation, and where his false beliefs about himself, life, others, and even God resided. In this way, he discovered his place in the world when he was admitted to the Royal Hospital for apparent insanity:

*"As he beheld the punishments meted out to the afflicted, who, like himself, were deemed insane, he said: May Jesus Christ grant me in due time the grace to establish a hospital where I may gather the poor, the helpless and those lacking sound judgment, that I might serve them as I desire"*^12^

In his past, John of God struggled with the powers of the soul, which his enemies disordered, making him a slave to thoughts, emotions, or impulsive actions^21^. Avila taught that the first step toward connecting with the *self* is self-knowledge, allowing a person to re-educate and perfect themselves in pursuit of integrity. However, he warned that self-deception is the greatest obstacle to this process, even in moments of vulnerability. Because of how they were raised, people may think they know who they are, but reality imposes itself, creating internal conflicts^17^. This is why John of God, through his spiritual care, was able to transition from seeing himself as a savior to the role of a caregiver, making it easier for others to accept and cope with the sufferings of others and their own suffering.

At the same time, Master Avila also recommends observing and understanding the virtues a person possesses, as this, too, is a form of humility. By doing so, people can be who they truly are and transcend themselves. One of the means that Master Avila suggests is meditation, especially on one's own mortality and that of loved ones. He also recommends seeking spiritual guidance, as having an external professional can more easily and confidently help dismantle beliefs about oneself than relying on one's own cognition^17^.

Although St. John of God was a nurse rather than a spiritual director, he could be considered a facilitator or a spiritual companion. He was always ready to support others through both works and words, fostering their spiritual growth and helping them reconnect with their selves or referring them to experts. For this reason, he institutionalized the ritual of the Catholic sacrament of confession in his hospital as a means of guided self-examination^12^. Nevertheless, Avila and John of God emphasize the progressive nature of the development of spiritual health due to the invisibility of the unconscious mechanisms that conceal the need for health:

*"But be ye warned, this business is not accomplished in a short time [...] that which is spoken in a word is fulfilled over many years"*^16^

In this way, people can be themselves, living in the truth, because "there is no greater deception than to be deceived in one’s own self, mistaking oneself for something other than what one truly is"^17^.

### 1.2 Relationship with others

For one's self to enter into a healthy relationship with another self, it is necessary to be fair, as St. John of God says, by "*giving each person what is theirs"*^14^. However, one is not always fair with others, nor are others fair toward oneself, nor are the collectives that act as a whole, such as families or communities, always fair to the individual. These groups may lack good *spiritual health,* exhibiting certain symptoms of low spiritual intelligence, such as dogmatism, fanaticism, or gregariousness^22^. According to Benavent^23^, *community spiritual health* is based on the common good, sustainability, wisdom, and holistic spirituality; it transcends the individual by contemplating it within the whole human ecosystem.

*"We are all one heart and one body, called to one faith and one inheritance, nourished with one bread and redeemed by one price; and where there exists such unity, there is but little division of the lands"*^16^

Each person is equal to every other person, according to his or her nature and vulnerability, although there may be differences among them, which is a cultural richness since the whole is greater than the parts^22^, so cultural awareness is necessary:

*"In all that thou behold in thy neighbor, see what thou dost feel, or wouldst desire others to feel concerning thee, were it to happen unto thee; and with the eyes that pass by thee, have compassion upon him, and remedy his plight as much as thou art able; and thou shalt be God’s measure with this pious measure by which thou measurest"*^17^

What today is conceived as empathy was already advocated in the 16th century, alongside compassion for others. As Lévinas stated, “I am insofar as the other is.” ^24^ Otherwise, the phenomenon of exclusion occurs, leading to a sense of uprooting, evident in individualistic Western societies that are afraid of those who are different, much like in the Granada of John of God, where Moors and converted Jews were despised and rejected^25^. While some accumulated wealth and honors, the rest lived on the brink of poverty, as seen in health care:

*"They have died while awaiting delays and decisions to be received. For some, there is a bed, while for others, there never is. This witness knows of this through his own sight in this city, in the hospitals of Charity, Veracruz and Corpus Christi, as well as in another that he does not name"*^15^

In contrast, John of God defended the disinherited and the different, considering them irreplaceable cells of the community, as can be deduced from his episodes with prostitutes, the sick, the marginalized, Muslims, and women. He is not afraid because he wins by increasing the whole. Because of his self knowledge, he considers others as equal in nature to himself, unlike those who despise them for their appearance:

*"The poor who are in the hospital are good, and I know of no vice within them; since God doth suffer both the wicked and the good spreading His sunshine upon all every day, it would not be just to cast the destitute and afflicted from their own abode"*^12^

As his spiritual director taught him:

*"For if Christ doth dwell with in thee, thou wilt feel about things as He felt, and thou shall see how rightly thou art bound to suffer and love thy neighbors, whom He loved and esteemed as the head loveth his body, and as the husband loveth his wife, and as the brother loveth his brother, and as a loving father his children”* ^17^

Moreover, John of God does not impose any truth, nor does he claim to possess it. He did not pretend gregariousness where everyone was equal through imitation; instead, he promoted the participation of all in his project without diluting the individual in the collective, respecting each person's vocation^14^. As Avila rightly said: "*Seek that way of life which shall most assure thy salvation, and not that which shall bring thee the greatest honor in the eyes of men*"^16^, just as John of God respected the vocation of Luis Bautista, Antón Martín, or those women he rescued by finding them a job, a husband or a religious life. In a society dichotomized between head and heart, John of God fostered values of altruism - connection with others- and a realistic hope for the world so that he tried to unite the opposites, as Hawks rightly observes^5^.

Thus, we could summarize spiritual health in terms of John of God’s relationship with others, showing empathy when he "looks with eyes that pass through himself and through Christ"^17^:

*"It is not possible to say or write, the profound love that is stirred in the heart of a Christian who behold his neighbors not according to outward appearances, such as wealth or lineage, or other such things, but as dear members of the body of Jesus Christ, most intimately united to Christ with all manner of kinship and friendship."* ^17^

### 1.3 Relationship with the environment

Currently, there is a disconnect from the environment due to a lack of responsibility in its care; the environment is treated as a tool, with its inherent respect and dignity being lost. However, the enjoyment derived from this utilitarianism is fleeting and leads to enslavement through attachment to the sensible, as Avila notes. Instead, he urges an authentic contemplation of nature that transcends it^17^. Nevertheless, Avila's perspective does not explicitly present an ecological theology; yet, an undercurrent of it can be observed when he speaks of creation, a contemplation of creation to find only beauty, just as beauty is found within the self. For him,

*"God is in everything created by essence, and presence, and power,"* ^16^ *and the creatures "speak loudly that God loves us well"* ^16^ *, "because in everything, as in an image and retable, the omnipotence and wisdom and goodness of the one who made it shine forth"*

^16^*:*

*"For if be not deaf, what is life, health, bread, wine, earth and heaven, yea, all in which we live and move and have our being, but voices that proclaim of the love you have for us and require of us?"* ^16^

In the face of life’s contingencies, John of God also appreciated the gift of life in nature, having been in contact with it throughout his childhood and youth. Although he always placed people above creation, he regarded nature as a necessary good in the ecosystem, forming part of life macrosystem, and saw himself as part of the whole:

*"When he was in the house of the Count of Oropesa, he beheld in the stable the horses, fat, sleek, and well-housed, while the poor were lean, naked and ill-treated; and he said within himself: “How now, Ioan, would it not be better for thee to tend and feed the poor of Jesus Christ than the beasts of the field?"* ^12^

Similarly, we could refer to the *self-body* relationship. If a person becomes dichotomized between the self and the environment, they also become dissociated between the spiritual and the corporeal self, losing their wholeness^26^. The ancient Hippocratic Greeks already spoke of a balance between body and soul, as Avila rightly points out:

*"Even as physical exercise is necessary to preserve the health of the body, so too is the exercise of the spirit necessary to preserve the health of the soul"*^16^

This unity is well understood by St. John of God, who recommends comprehensive care:

*"In three matters, good Duchess, thou must dedicate the time of each day: in prayer, in labor, and in the care of the body"*^14^

It is well known that a healthy spirituality encourages individuals to take responsibility for their self care. The individuals would respect the body and its needs while also being informed about what is harmful to them. By listening to the body, the spirit would be listened to and vice versa^27^, as Avila rightly says:

*"And take heed not to afflict thine heart with forced sorrow [...] for such grief doth tend to destroy the health of the body”* ^17^

This relationship between spiritual suffering and physical illness can be seen in Renaissance society:

*"By reason of the great sorrow they bore from being exiled and impoverished, they all fell ill"*^12^

St. John of God himself tells us how sadness worsened his health at the end of his life:

*"Among the destitute folk who came to gather firewood, a young lad, incautious, ventured into the river beyond his strength, whereupon the current did seize him and carry him away. And John of God, to aid him, entered the waters far beyond, but the lad did drown, for he could not hold fast to him. This caused him great sorrow, and thus his ailment did worsen every day"*^12^

Certainly, in Christian spirituality, a certain contempt for the body can be misinterpreted as asceticism; however, in this spirituality, these practices are considered a tool for the education of the passions, and always in moderation, as Avila recommends:

*"I am wary of your worship, sir, and of the licentiate even more so, for it is certain that some excesses in penance hath brought on this malady. And where it that thou art ill, I would rebuke thee sternly; yet thou shalt recover, and the correction shall come in due time. For self-will must be curbed no less in matters deemed good than in those which are plenty not, for it is self-will itself that turneth even good things to ill"*^16^

This lack of self-care due to a false idea is evident in the spirituality of individuals with anorexia nervosa; in spiritual health, the body is embraced as part of the *self,* whereas in pathological conditions, the body is rejected. In this way, spiritual health could contribute to self-care and body awareness as the body is an integral part of the person, though its needs should be prioritized appropriately.

### 1.4 Relationship with the transcendent world

Every person needs a sense of meaning to guide their life, but this is ever-changing, and it is up to the person to discover it at each moment. However, while every action needs a meaning, a person also needs a "supra-meaning" that encompasses their entire existence. This deeper purpose points to this domain and is the ultimate meaning of existence, historically identified with God. It is not that God grants meaning to existence; rather, as Frankl suggest, the person can discover meaning through faith in Him^28^. Therefore, religion would serve as a mechanism that aids individuals in their search for ultimate meaning by promoting existential experiences, not through reason^29^, as the mysticism of the Spanish Renaissance rightly points out. That is why Avila says:

*"And be it known that this matter is more of the heart than of the head, for to love is the end of thought. And not for understanding of this, nor of the peace afore spoken, have many heads been wearied, their own and others’, bringing harm to their health and hindrance to the good they might have wrought"*^16^

In this way, as Frankl^29^ points out, it is through faith that one gains access to this ultimate supernatural meaning; however, this faith is based on experience, that is, on the love that transforms the person and becomes a lived reality to be believed intellectually, as a personal adherence to a being that cannot be seen with the senses yet is all-encompassing. Avila says:

*"But if we are content to know God by faith, and not by that experimental message born from love, and follow after human conjectures, we shall likewise have reason to weep as he did and say: Woe unto the time when I loved Thee not!"* ^16^

However, the relationship with the transcendental is not always healthy, as seen in cases of fundamentalism. When only dogmas or rational truths are upheld, it is for no other reason than the fear of emptiness or the risks of existence. This is also true for the sick, who consider illness as a divine punishment^30^, which stands in stark contrast to what Avila or St. John of God proposed. Thus, if the transcendental relationship is not based on the experience of love, it cannot be salutogenic, as Frankl^29^ points out. It is through love that the person accepts existence and can enter the depths of the previous domains. Therefore, a person who is loved can transcend by loving others, becoming part of the totality of existence. St. John of Avila says:

*"She doth not rest until, by conjecture, she feelth herself to be both loved and loving; for until an anima doth feel this, she liveth ever in fear, sorrow, and the burden of the law. But when she hath attained thereto, naught may lightly trouble her, for she deemeth that God is with her and she is in God"*^16^

When people are loved, they can surrender to existence in all its circumstances, loving in return for being loved, which Avila refers to as Redamation:

*"The more this Lord is conversed with, the more shall He be known, and the more He is known, the more shall He be loved"*^16^

John of God expresses the materialization of his master’s theory of Redamation as follows:

*"If we did but see how great the mercy of God is, we would ne’er cease from doing good as long as we could, for in giving for His love what He hath bestowed upon us, He doth promise us a hundredfold in blessedness. O, blessed gain and usury! Who would not render unto this most blessed Merchant all that he hath, since He maketh with us such a good bargain and beseeched us with open arms to repent and weep for our sins, and to do charity, first to our souls and then to our neighbors, for as the soul slay the fire, so doth charity slay sin"*^14^

St. John of God draws from his own experience of love, which becomes a gift to others, loving them in return. In particular, he urges self-transcendence because, even if people are not sick, if they do not live accepting and contemplating their necessary being, they can suffer. However, love whether divine or for other universal reasons, decenters individuals and orders their passions and powers toward the common good, harmonizing with the individual good.

Consequently, people are not conceived for what they are but for what they can become, for what their faith promises them:

*"It is love alone that quickeneth all things, and it is the very spiritual remedy for our anima, without which she is as the body is without her"*^16^

In this sense, as Frankl29 points out, John of God and John of Avila served through their care, healing people's wounds with love and being salutogenic for individuals and communities so that they transcended their reality and reached a place where they could *"give thanks for what is understood and for what is not understood, for therein lies health*"^16^.

### Limitations of the study

Although pertinent information can be gathered from documentary sources, it is important to note that most of the texts about St. John of God are secondary sources. While these sources are highly reliable, particularly as they are legal documents from the period in question, it remains essential to approach them cautiously and adhere to the principles of prudence.

### Practical applications

The findings of this study suggest that the health outcome according to the NOC Spiritual Health could be validated in future research because, as observed, it aligns with the practical criteria currently recognized in nursing. It may serve as a model for delivering comprehensive care through this dimension of the human being. 

## Conclusion

The concept of spiritual health was already well-established in 16th-century Spain, particularly within the field of nursing. St. John of God, the reforming father of Spanish nursing (1495-1550), and his spiritual master, St. John of Ávila, viewed health as a continuum between what a person is and what they can become. It is a path to achieving fullness and wholeness of the individual, the family, and the community. It begins with self-knowledge, enabling a person to uncover the truth about themselves, their needs, and obstacles and to foster a relationship with themselves. From this foundation, the goal is to seek connection with others, building communities and relationships based on values that aimed at the common good. It also involves a balanced relationship between the natural world and the individual, encompassing the intangible realm of the body. Ultimately, spiritual health encompasses the search for meaning with God as the dominant interpretation of meaning in this period and the source of love that fosters individual and collective peace through comprehensive development.
